# Environmental factors influencing *Culicoides* vectors of Bluetongue in northwestern Spain: abundance, phenology and epidemiological risk

**DOI:** 10.1186/s13071-026-07453-7

**Published:** 2026-05-28

**Authors:** Alejandro Polina, Yasmina Martínez-Barciela, Ignacio Ruiz-Arrondo, Rosa Estrada, Josefina Garrido

**Affiliations:** 1 Departamento de Ecoloxía e Bioloxía Animal, Facultade de Bioloxía, Universidade de Vigo, Vigo, Spain; 2 Department of Animal Pathology, Faculty of Veterinary Sciences, Instituto Agroalimentario de Aragón-IA2 (Universidad de Zaragoza-CITA), Zaragoza, Spain

**Keywords:** Environmental variables, Biting midges, Obsoletus complex, *Culicoides punctatus*, Vector activity period, Veterinary health, Galicia, Basic reproduction number, Epidemiological risk

## Abstract

**Background:**

The genus *Culicoides* comprises several vectors of the Bluetongue virus (BTV) affecting livestock and other ruminants. The historical occurrence and recent re-emergence of this disease in Galicia (NW Spain) make it essential to identify the vector species present in the region, determine the factors influencing their abundance, analyse their seasonal activity period and identify areas of highest epidemiological risk.

**Methods:**

A total of 2009 light-trap collections targeting *Culicoides* were conducted at representative sites across Galicia between 2008 and 2012. A subset of the 2009 dataset was used to analyse species phenology and epidemiological risk. Several climatic and environmental factors were obtained from different sources to determine their relationship with the abundance of the identified potential BTV vectors through Negative Binomial Generalized Linear Models (NBGLMM).

**Results:**

Five potential BTV vectors were identified in the region: *Culicoides obsoletus* s.l., *C. punctatus*, *C. newsteadi*, *C. pulicaris* and *C. imicola*. NBGLMM provided a good fit for the Obsoletus complex (*R*^2^c = 0.68) and for *C. punctatus* (*R*^2^c = 0.54). Non-linear correlations were observed between Obsoletus complex abundance and environmental variables. High temperatures 6 months prior to the collection date were associated with a decrease in the abundance of the Obsoletus complex, whereas higher mean minimum temperatures 28 days prior to sampling, NDVI and seasonality had a positive impact. *Culicoides punctatus* abundance was negatively correlated with higher mean maximum temperatures 6 months before sampling, while higher mean minimum temperatures 2 months prior to collection, altitude and Csb-type climate were positively associated. The vector activity period (VAP) averaged 23.9 ± 6.0 weeks per year. The deterministic basic reproduction number *R*_0_ indicated a potential BTV transmission risk period of 16.5 ± 7.0 weeks annually, whereas the Monte Carlo approach estimated a period of 14.8 ± 5.6 weeks per year. This means a period approximately 31.0–38.1% shorter than the VAP, suggesting that periods of vector presence do not necessarily translate into sustained disease transmission potential.

**Conclusions:**

This research provides valuable insights into the ecological determinants of BTV vectors abundance under Atlantic climate conditions. These findings are crucial for understanding disease transmission dynamics and improving vector control strategies. By integrating climatic characteristics and identifying areas of highest epidemiological risk, public health interventions and management measures can become more targeted and efficient.

**Graphical Abstract:**

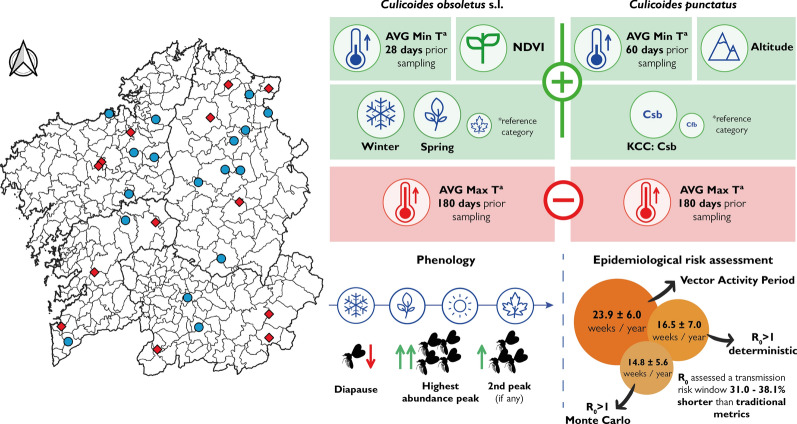

## Background

The genus *Culicoides* Latreille (Diptera: Ceratopogonidae) comprises a wide variety of species of biting midges capable of transmitting several diseases of veterinary concern, such as Bluetongue virus (BTV), Schmallenberg virus (SBV), and Epizootic Haemorrhagic Disease virus (EHDV), the latter having been recently introduced into Europe via Italy and Spain [1, 2]. BTV belongs to the genus *Orbivirus* (Reovirales: Reoviridae) and comprises 29 different serotypes with diverse clinical implications for both livestock and wild ruminants, including new putative serotypes identified in recent years [3, 4]. Infection with BTV in livestock has a considerable socioeconomic impact not only through morbidity and mortality but also by triggering trade restrictions aimed at containing its spread [5–7].

In Spain, the disease was considered eradicated from 1958 until its re-emergence in 2000, when the BTV-2 serotype was detected in the Balearic Islands [8]. In 2004, the BTV-4 serotype was found circulating in Cádiz, in southern mainland Spain, from where it rapidly spread to other regions [9]. Its proliferation led to several outbreaks across the country, resulting in substantial economic losses [7]. This situation also affected northern regions, and some authors have suggested windborne dispersal of infected vectors as a probable mechanism for virus introduction [10]. Galicia, located in northwestern Spain, was no exception: the first cases reported in 2007 were associated with the circulation of the BTV-1 serotype [11]. After successful vaccination campaigns, BTV was considered under control in the region until 2023, when several outbreaks of the BTV-4 strain were detected in sentinel farms [12]. Subsequently, serotypes 3 and 8 were detected in 2025, spreading to most regional municipalities [13]. The re-emergence of viral circulation is of particular concern, as agriculture and livestock farming are key drivers of the region’s economic development.

Since 2004, all Autonomous Communities have participated in the Bluetongue National Surveillance Program (hereinafter referred to as PNVLA, by its Spanish acronym), established by the Ministry of Agriculture, Fisheries and Food (MAPA, by its Spanish acronym). The program is structured around three main components: (i) active serological and virological surveillance; (ii) passive clinical surveillance; and (iii) entomological monitoring and surveillance. The present study focuses exclusively on the third component. This pioneering multidisciplinary surveillance program was implemented prior to the European Union (EU) Regulation 1266/2007, which recommended the establishment of large-scale entomological monitoring networks for the EU members [14]. The PNVLA aimed to determine the annual fluctuations, distribution and temporal abundance of populations of the species considered as vectors, namely *Culicoides imicola* Kieffer*, Culicoides obsoletus* (Meigen) s.l. – recent taxonomic studies only include *Culicoides obsoletus* (Meigen) s.s. and *Culicoides scoticus* Downes & Kettle within this complex [15] – and *Culicoides pulicaris* (Linnaeus) s.l.. On the basis of these data, the vector seasonally free periods (VFP) can be estimated for each region each year [16]. Vector-free periods are declared annually on the basis of entomological capture records.

Extensive sampling has been carried out under the PNVLA throughout the country until now [17, 18]. However, data from several Autonomous Communities have yet to be published, as is the case for Galicia. Although some studies have addressed the distribution and veterinary-sanitary relevance of *Culicoides* species present in Galicia [16, 19, 20], there remains a need to increase knowledge of the seasonal dynamics of their populations and the environmental drivers influencing their proliferation. This is an essential step toward establishing effective preventive measures for host protection and vector control [21–23].

Therefore, the objective of the present study is to examine the relationship of different biotic and abiotic factors on the abundance and distribution of potential BTV vectors in Galicia, as well as to determine their activity periods and to assess the epidemiological risk in representative areas of their development.

## Methods

### Study area

The study was conducted in the Autonomous Community of Galicia, in north-western Spain, which covers an area of 29,579 km^2^ (5.8% of the national territory). The region encompasses a wide range of altitudes, from sea level to over 2000 m. According to the Köppen climate classification (KCC), two main climate zones can be identified: Cfb (oceanic temperate) in the northern and some western areas; and Csb (oceanic Mediterranean) in the central and southern area. A third, much smaller zone (Csa–typical Mediterranean) is restricted to certain areas along the southern border, but no sampling points were established there. In the Cfb climate zone, the mean temperature of the warmest month never exceeds 22 °C, although there are at least 4 months with a mean temperature above 10 °C. Rainfall is evenly distributed throughout the year. The Csb zone shares similar temperature patterns, but its summers are significantly drier, and precipitation is more irregular over the course of the year [24, 25].

### Sampling procedure

**Fig. 1:**
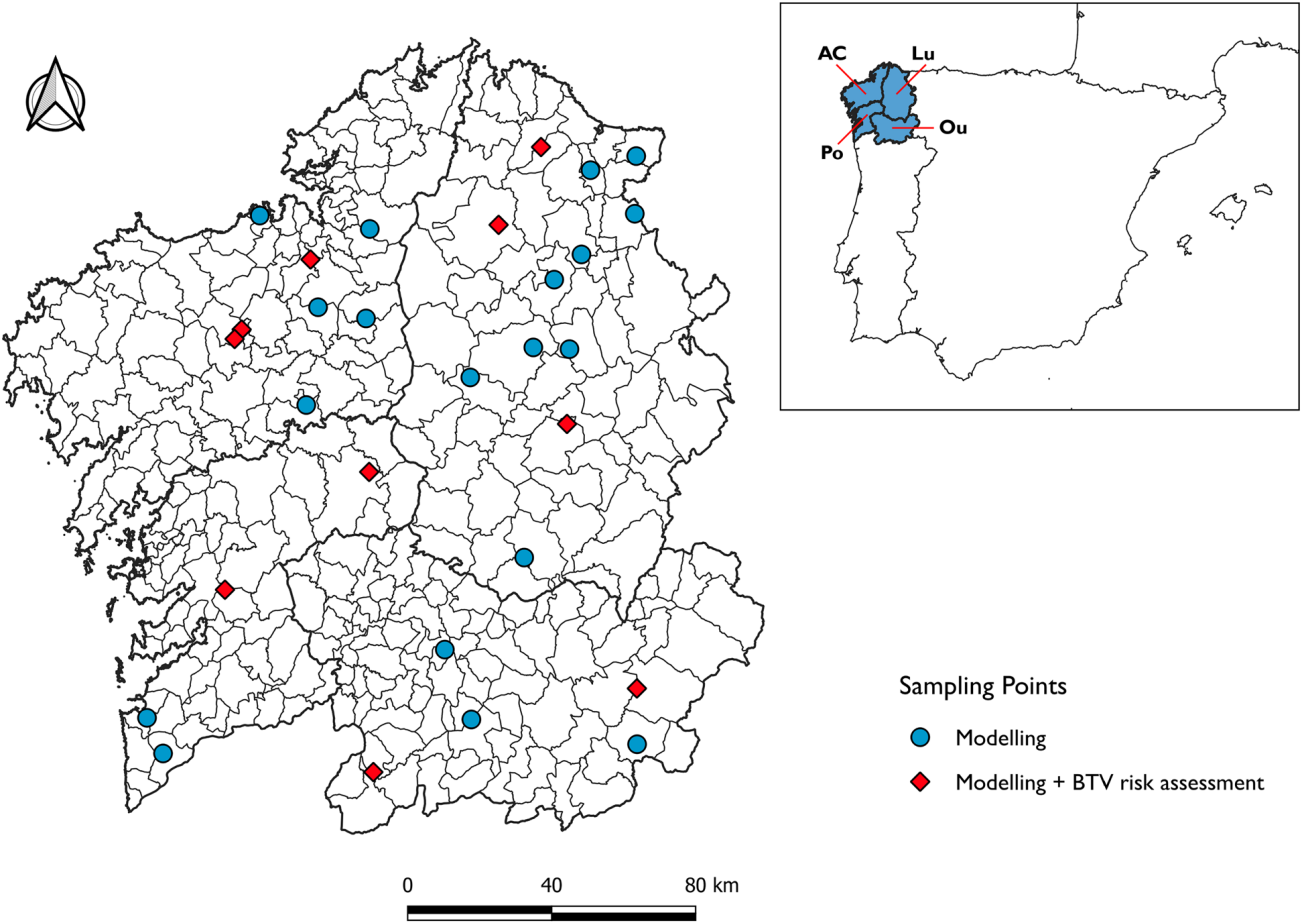
Study area showing the sampling points deployed across the Autonomous Community of Galicia. Municipal boundaries (NUTS 3) are also shown. Top right: location of the region and its provinces within the Iberian Peninsula. Blue-circled sites were used to assess modelling performance, whereas red-squared sites were additionally analysed to characterise phenology, vector activity periods and basic reproduction numbers. *AC* A Coruña province, *Lu* Lugo province, *Ou* Ourense province, *Po* Pontevedra province

The data were gathered under the PNVLA framework, funded by the Spanish Government from 2004 to the present. The dataset analysed in this study covers the 2008–2012 period in Galicia, when surveillance efforts were intensified due to BTV outbreaks in the region. This period was selected as the corresponding data is more complete and cover a large portion of the region, which improves the accuracy of the models. A total of 30 sampling sites distributed across the four Galician provinces were monitored: 8 in A Coruña, 12 in Lugo, 5 in Ourense and 5 in Pontevedra (Fig. 1).

A subset of the data was used to analyse species phenology to ensure adequate sampling effort throughout the year. Following the criteria proposed by Barceló et al. [23], only site-years with at least 44 trapping weeks and no more than three consecutive weeks without trapping were included. Based on these requirements, 10 locations were selected for 2009.

Sampling points were selected in areas considered suitable for *Culicoides* occurrence, such as livestock farms and slaughterhouses. CDC miniature light traps (John W. Hock Company) equipped with 4 W UV bulbs and collector bottles containing antifreeze and alcohol to preserve specimens were used for capture. Several sentinel sites were sampled weekly from January to December, while others were sampled irregularly throughout the season. The traps were hung at a height of 1.5–1.8 m inside stables and operated for 24 h. Collection bottles were transported to the reference laboratory at the Faculty of Veterinary, University of Zaragoza. When possible, specimens were identified to the species level using the morphological identification key provided by Mathieu et al. [26]. Females of species within the Obsoletus complex cannot be reliably distinguished on the basis of morphological features and were therefore classified at the complex level. In this study, the Obsoletus complex comprises *C. obsoletus* s.s., *C. scoticus*, *Culicoides chiopterus* (Meigen), *Culicoides dewulfi* Goethebuer and *Culicoides montanus* Shakirzjanova, following the grouping applied in the surveillance framework during the study period. Females were further categorised according to their gonotrophic state (nulliparous, parous, blood-fed and gravid). The resulting data were sent to the Laboratory of Entomology at the University of Vigo for subsequent analysis.

### Environmental and host variables

The variables analysed in the present study are shown in Table 1. Climatic data included mean temperature; maximum and minimum temperatures; mean maximum and mean minimum temperatures; accumulated and mean rainfall; and mean relative humidity. These parameters were calculated for periods of 7, 14, 21, 28, 45, 60, 90, 120, 150 and 180 days prior to each sampling date. Data were collected from the nearest meteorological station at a similar altitude, available through the MeteoGalicia website (https://www.meteogalicia.gal/observacion/estacionshistorico/historico.action?request_locale=es), a service managed by the Xunta de Galicia. Wind speed could not be included because most meteorological stations did not record this variable during the analysed period.

**Table 1:** Variables included in the present study, showing the codes for each one

Category	Variable	Code
Climatic	Accumulated rainfall prior to the sampling date (°C)	X7SUM_PREC, X14SUM_PREC, X21SUM_PREC, X28SUM_PREC, X45SUM_PREC, X60SUM_PREC, X90SUM_PREC, X120SUM_PREC, X150SUM_PREC, X180SUM_PREC
	Average rainfall prior the sampling date (°C)	X7AVG_PREC, X14AVG_PREC, X21AVG_PREC, X28AVG_PREC, X45AVG_PREC, X60AVG_PREC, X90AVG_PREC, X120AVG_PREC, X150AVG_PREC, 180AVG_PREC
	Maximum temperature reached prior the sampling date (°C)	X7MAX_TMAX, X14MAX_TMAX, X21MAX_TMAX, X28MAX_TMAX, X45MAX_TMAX, X60MAX_TMAX, X90MAX_TMAX, X120MAX_TMAX, X150MAX_TMAX, X180MAX_TMAX
	Minimum temperature reached prior the sampling date (°C)	X7MIN_TMIN, X14MIN_TMIN, X21MIN_TMIN, X28MIN_TMIN, X45MIN_TMIN, X60MIN_TMIN, X90MIN_TMIN, X120MIN_TMIN, X150MIN_TMIN, X180MIN_TMIN
	Average maximum temperature prior the sampling date (°C)	X7AVG_TMAX, X14AVG_TMAX, X21AVG_TMAX, X28AVG_TMAX, X45AVG_TMAX, X60AVG_TMAX, X90AVG_TMAX, X120AVG_TMAX, X150AVG_TMAX, X180AVG_TMAX
	Average mean temperature prior the sampling date (°C)	X7AVG_TMEAN, X14AVG_TMEAN, X21AVG_TMEAN, X28AVG_TMEAN, X45AVG_TMEAN, X60AVG_TMEAN, X90AVG_TMEAN, X120AVG_TMEAN, X150AVG_TMEAN, X180AVG_TMEAN
	Average minimum temperature prior the sampling date (°C)	X7AVG_TMIN, X14AVG_TMIN, X21AVG_TMIN, X28AVG_TMIN, X45AVG_TMIN, X60AVG_TMIN, X90AVG_TMIN, X120AVG_TMIN, X150AVG_TMIN, X180AVG_TMIN
	Average mean relative humidity prior the sampling date	X7AVG_HMEAN, X14AVG_HMEAN, X21AVG_HMEAN, X28AVG_HMEAN, X45AVG_HMEAN, X60AVG_HMEAN, X90AVG_HMEAN, X120AVG_HMEAN, X150AVG_HMEAN, X180AVG_HMEAN
	Köppen climate classification	KCC/Csb
		KCC/Cfb
	Season	SEASON/Autumn
		SEASON/Winter
		SEASON/Spring
		SEASON/Summer
Landscape	Altitude (m)	ALT
	Normalised difference vegetation index	NDVI
	Land use	LAND_USE/Urban areas
		LAND_USE/Forests
		LAND_USE/Crops and grasslands
		LAND USE/Heath areas
Host	Type of host	Cows, small ruminants, horses
	Total density of hosts (km^−2^)	TOTAL_D
	Density of cows (km^−2^)	COWS_D
	Density of small ruminants (km^−2^)	SMALL_D
	Density of horses (km^−2^)	HORSES_D
Methodology	Distance between the trap and the nearest host	TRAP_DIST
	Type of place	PLACE_TYPE/Livestock farm
		PLACE_TYPE/Slaughterhouses

Codes for the qualitative variables are represented with their categorizations

Altitude values were obtained using Quantum GIS version 3.34 Prizren [27]. Köppen climate classification regions and land-use data for Galicia were obtained from the Instituto Geográfico Nacional (IGN) of the Spanish Government through its online portal (https://www.ign.es/web/ign/portal). The 20 landscape categories defined in Galicia were grouped into five main land-use types: wetlands, forests, heath areas, crops and grasslands and urban areas. No sampling sites were established in wetland areas, so this land-use type could not be considered in the study.

The Normalized Difference Vegetation Index (NDVI) provides information on the vegetation cover at a given location. It is calculated as the difference between near-infrared and visible red reflectance values divided by their sum. NDVI data were obtained from the Copernicus Land Monitoring Service of the European Union through its digital repository (https://land.copernicus.eu/en/products/vegetation) in Network Common Data Form (NetCDF) format. To extract data for the 2007–2013 period, global NDVI raster layers covering 1999–2020, with a spatial resolution of 1 km and averaged over 10-day periods, were used.

Host density was estimated by dividing the livestock census data provided by the regional government of the Xunta de Galicia for each farm by the farmhouse area. A dichotomous variable indicating the presence of each host type (cows, small ruminants–sheep and/or goats–, and horses) was also considered (1 = presence; 0 = absence).

### Data analysis

Prior to applying the most appropriate models to explain the abundance of the different species (Obsoletus complex, *Culicoides punctatus* (Meigen)*, C. pulicaris* and *Culicoides newsteadi* (Austen)) in relation to the environmental factors (biotic and abiotic) under study, the variables and overall behaviour of the data were examined. Pairwise correlation tests were performed to identify highly correlated variables (*r* > 0.7, *P* < 0.05) in order to remove redundant predictors. As a result, mean rainfall variables for different time periods were excluded from subsequent analyses.

The Shapiro–Wilk test was used to determine whether both predictor and response variables followed a normal distribution (*P* > 0.05). Variables deviating from normality were log-transformed to improve model performance. Given the strong overdispersion in the abundance data (variance greater than mean), a negative binomial generalized linear mixed model (NBGLMM) was fitted, including sampling point as a random effect and environmental variables as fixed effects, using the *glmer* function from the Matrix package in R software [28, 29]. Fixed effects represent population-level parameters consistent across observations, whereas random effects account for site-specific variability that may influence the response variable.

Model selection was performed using a forward stepwise approach based on analysis of variance (ANOVA) model comparisons (*P* < 0.05), retaining only statistically significant predictors from the model with the lowest Akaike Information Criterion (AIC). Variance inflation factors (VIFs) were examined to identify highly correlated predictors and avoid multicollinearity in the model (VIFs < 5). Finally, marginal *R*^2^ (*R*^2^m, proportion of variance explained by the fixed effects) and conditional *R*^2^ (*R*^2^c, proportion of variance explained for both fixed and random effects) were calculated to assess the explanatory power of the final models.

All statistical analyses were performed by using BiodiversityR, lme4, Matrix and MuMin packages of the R software version 4.4.2 [28–32].

### Vector activity period (VAP) and basic reproduction number *R*_0_

According to European Commission Regulation (EC) No 1266/2007 on the control, monitoring, surveillance, and movement restrictions of certain animal species susceptible to BTV, a seasonally BTV-free period is defined by the absence of virus transmission evidence and the occurrence of a seasonally vector-free period (VFP) [14]. The beginning of the transmission season is established when at least five parous females of species proven or suspected to be vectors of BTV in a given area are captured. The opposite concept would be the vector activity period (VAP), a term that is analysed in the present study. In Spain, the main vectors responsible for BTV transmission are *Culicoides imicola* in the southern regions and the Obsoletus complex in the north [16]. Since only two *C. imicola* specimens were collected during the sampling period in Galicia, only the dynamics of the Obsoletus complex and of *C. punctatus* were considered in the study.

As a complementary and more precise approach to quantify the risk of a potential BTV outbreak, the basic reproduction number *R*_0_ was calculated. This metric is based on the foundational work of Ross and Macdonald [33] and has been applied to several vector-borne diseases, including BTV and African horse sickness (AHS) [34–36]. *R*_0_ is defined as the number of secondary cases generated by a single infectious individual during its entire infectious period in a completely susceptible population. Therefore, if *R*_0_ > 1, the virus can invade the population and cause an epidemic, whereas if *R*_0_ < 1, the infection would fade out without further transmission [37].

The basic reproduction number equation can be read as follows:

$$R_{0} \, = \,\sqrt {\frac{{k\left( T \right)^{2} \,p_{M} \gamma_{M} \left( T \right)}}{{m_{M} \left[ {\gamma_{M} \left( T \right)\, + \,m_{M} \left( T \right)} \right]}}\,\frac{{N_{M} }}{{\left( {\sum\nolimits_{i} {N_{i} } } \right)^{2} }}\,\sum\limits_{i} {\frac{{p_{i} N_{i} }}{{\alpha_{i} \, + v_{i} }}} }$$

This basic reproduction number is partly temperature dependent and considers vector or virus specific parameters, vector abundance and host densities. Most rates were obtained through different studies carried out in the field or laboratory (Table 2) [35, 38–41]. Host densities (*N*_*i*_) were calculated by dividing the census by the farmhouse area, with cattle (C) and small ruminants (S) as the hosts involved in BTV. The vector density *(N*_*M*_) was estimated assuming that trap collections represent 1% of the local vector population [35]. Only specimens belonging to the Obsoletus complex were included in the calculation of this index.

**Table 2:** Parameters included in the basic reproduction number equation (*R*_*0*_)

Parameter	Symbol	Value/Function	Refs.
Vector biting rate	k(T)	0.00017T (T−3.70) (41.87−T)^1/2.71^	[38]
Virus reproduction rate in vector	*γ* _*M*_ (T)	0.017(T−12.6)	[39]
Vector mortality rate	*m* _*M*_ (T)	0.0089 exp (0.155T)	[40]
Transmission probability vector to host	*p* _*M*_ for BT	1.000	[35]
Transmission probability host to vector	*p* _*C*_ = *p* _*S*_ for BT	0.050	[35]
Removal rate of hosts	*α* _*C*_ for BT	0.055	[41]
	*α* _*S*_ for BT	0.125	[35]
Fraction dying due to infection	*ν* _*C*_ = *ν* _*S*_ for BT	0.000	[35]

Symbols and values/functions applied for Bluetongue (BT) are shown

Parameter uncertainty was explored using a Monte Carlo simulation approach [42]. For each sampling event, 5000 simulations were performed for each key vector-related parameter – vector biting rate, virus reproduction rate and vector mortality rate – from normal distributions with a coefficient of variation of 20% to reflect plausible biological variability. The resulting distribution of *R*_0_ values was used to estimate the mean and 95% uncertainty interval (2.5–97.5 percentiles). Simulations producing non-finite values were discarded, as temperature-dependent parameters resulted in conditions incompatible with virus transmission. Therefore, the resulting *R*_0_ values were set to zero. All analyses were conducted using the statistical environment R, version 4.4.2 [28].

## Results

### Abundance and frequency of species

A total of 60,926 *Culicoides* specimens belonging to 14 different species were identified in the present study. Five species are recognised as confirmed or suspected vectors of BTV: *Culicoides obsoletus* s.l., *C. punctatus*, *C. pulicaris*, *C. newsteadi* and *C. imicola*. Their relative abundances were highly uneven, with 66.85% of all specimens belonging to the Obsoletus complex. *Culicoides punctatus* and *C. pulicaris* were the second and fourth most abundant species, accounting for 12.04% and 5.43% of all individuals, respectively. *Culicoides achrayi* was the third most abundant species; however, it is not considered to have epidemiological relevance for disease transmission. *Culicoides newsteadi* was present at low abundance (0.48%), and *C. imicola* was nearly absent, with only two individuals collected (Table 3).

**Table 3:** List of species identified during the 2008–2012 monitoring campaign

Subgenus	Species	*N*	*N*’(%)	*F*	*F*’(%)
*Avaritia* Fox, 1955	*imicola* Kieffer, 1913	2	0.003	2	0.10
	*obsoletus* s.l. (Meigen, 1818)	40734	66.85	1152	57.37
*Beltranmyia* Vargas, 1953	*circumscriptus* Kieffer, 1918	74	0.12	8	0.40
*Culicoides* Latreille, 1809	*impunctatus* Goethebuer, 1920	5	0.01	4	0.20
	*newsteadi* Austen, 1921	293	0.48	22	1.10
	*pulicaris* (Linnaeus, 1758)	3311	5.43	107	5.33
	*punctatus* (Meigen, 1818)	7333	12.04	465	23.16
*Monoculicoides* Khalaf, 1954	*nubeculosus* (Meigen, 1830)	2079	3.41	8	0.40
	*parroti* Kieffer, 1914	3	0.005	3	0.15
*Oecacta* Poey, 1853	*brunnicans* Edwards, 1939	1512	2.48	40	1.99
*Sensiculicoides* Shevchenko, 1977	*festivipennis* Kieffer, 1914	397	0.65	45	2.24
	*maritimus* Kieffer, 1924	43	0.07	4	0.20
	*univittatus* Vimmer, 1932	830	1.36	30	1.49
*Silvaticulicoides* Glukhova, 1972	*achrayi* Kettle & Lawson, 1955	4315	7.08	117	5.83

*N* total number of individuals per species, *N’* percentage of individuals of each species over total, *F* number of samples positive for each species, *F’* percentage of samples positive for each species over total

Among the potential BTV vectors, the Obsoletus complex was by far the most frequent, occurring in 57.37% of the samples analysed. It was followed by *C. punctatus* (23.16%) and *C. pulicaris* (5.33%). *Culicoides newsteadi* was detected in only 1.10% of the samples and *C. imicola* in just two (0.10%) (Table 3).

### Performance of models

Models were performed for all potential BTV vectors except for *C. imicola,* owing to its very low abundance and restricted distribution (Table 4). The fitted NBGLMM for the Obsoletus complex yielded a *R*^2^c = 0.68, indicating that both fixed and random effects together explained a substantial proportion of the variance in abundance. Four predictors significantly influenced this taxon: NDVI, x28AVGTmin, and SEASON had positive effects, whereas x180AVGTmax had a negative impact (Table 4).

**Table 4:** NBGLMM obtained for the abundance of the potential BTV vectors in Galicia

Species	NBGLMM statistics				Performance measures		
	Predictor	*β* ± SE	*Z*	*P*	AIC	*R*^2^m	*R*^2^c
*C. obsoletus* s.l	Intercept	−1.41 ± 1.60	−0.88	0.38	11177.5	0.51	0.68
	NDVI	1.76 ± 0.27	6.49	8.40 × 10^−11^			
	x180AVGTmax	−4.00 ± 0.51	−7.83	4.94 × 10^−15^			
	x28AVGTmin	3.92 ± 0.40	9.77	< 2 × 10^−16^			
	SEASON [T.Autumn]	Reference category					
	SEASON [T.Spring]	0.67 ± 0.25	2.68	0.73 × 10^−2^			
	SEASON [T.Summer]	0.19 ± 0.18	1.03	0.30			
	SEASON [T.Winter]	1.22 ± 0.23	5.26	1.52 × 10^−07^			
*C. punctatus*	Intercept	0.05 ± 1.98	0.02	0.98	4496.3	0.49	0.54
	x180AVGTmax	−10.51 ± 0.55	−19.16	< 2 × 10^−16^			
	x60AVGTmin	4.92 ± 0.54	9.18	< 2 × 10^−16^			
	Alt	1.03 ± 0.31	3.21	0.79 × 10^−3^			
	KCC [T.Cfb]	Reference category					
	KCC [T.Csb]	2.04 ± 0.63	8.15	0.13 × 10^−2^			
*C. pulicaris*	Intercept	−29.71 ± 4.74	−6.26	3.84e^−10^	1385.9	0.19	0.22
	x14MAXTmax	7.25 ± 1.40	5.19	2.12 × 10^−07^			
	Alt	0.96 ± 0.37	2.57	0.01			

Predictors with their correspondent coefficients are shown (*β* parameter estimates, *SE* standard error, *Z* *z*-value, *P* *P*-value), as well as the Akaike Information Criterion (AIC), marginal *R*^2^ (*R*^2^m) and conditional *R*^2^ (*R*^2^c) obtained for the final models

The model predicting *C. punctatus* abundance showed a *R*^2^c = 0.54, also indicating an acceptable fit. Four predictors were retained in the final model: x60AVGTmin, Alt, and KCC, which had positive effects, while x180AVGTmax had a strong negative impact (Table 4).

Models fitted for *C. pulicaris* and *C. newsteadi* exhibited poor fits (*R*^2^c < 0.22 and *R*^2^c < 0.05, respectively). Nevertheless, the model for *C. pulicaris* indicated that x14MAXTmax, in combination with altitude (Alt), had a strong influence on species abundance, explaining approximately 19% of the variability (Table 4). No improvement in model performance was observed by adding other predictors. Overall abundance patterns of *C. newsteadi* were poorly explained by the tested variables, as none were statistically significant, preventing the establishment of a reliable predictive model.

### Phenology and epidemiological risk assessments

**Fig. 2:**
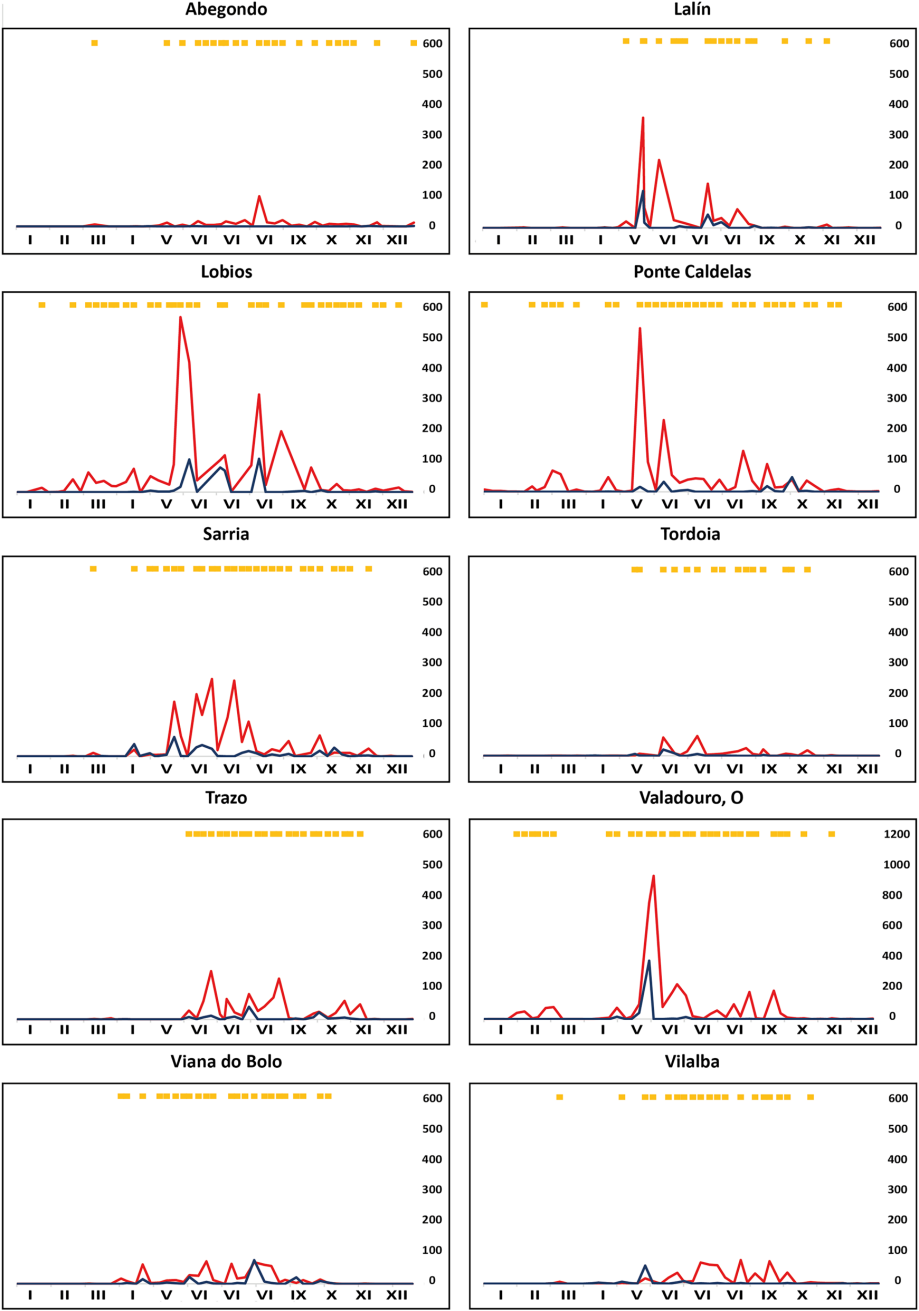
Temporal dynamics of captures of the two most dominant potential BTV vectors across the 10 study sites during 2009. Red line: variation of the captures of the Obsoletus complex; blue line: variation of the captures of *C. punctatus*. Yellow squares indicate the VAP as defined by the Spanish Commission Regulation (EC) No. 1266/2007

*Culicoides obsoletus* s.l. and *C. punctatus* were the only two species of BTV vectors present in all 10 sites sampled during the whole year, with the former being the most abundant at all sites. Their annual cycle showed very low numbers for both species during late autumn and winter months, while maximum abundances were observed in late spring and early summer, including May, June and July. Regarding sampling sites, higher abundances were recorded in Lalín, Lobios, Ponte Caldelas and O Valadouro, with the latter registering the highest peak for the Obsoletus complex (*n* = 929) and for *C. punctatus* (*n* = 379), both in June (Fig. 2). On the contrary, the lowest number of specimens were recorded in Abegondo and Tordoia, both located in the A Coruña province (Fig. 2). No clear seasonal peak of abundance was observed at some sites, such as Tordoia or Viana do Bolo (Fig. 2).

Considering these 10 site-by-year combinations, VAP lasted for 23.9 ± 6.0 weeks per year (approximately 167 days a year). Nevertheless, there were some noticeable differences among locations. Lobios, Ponte Caldelas and O Valadouro showed an almost continuous vector activity period throughout the year, whereas Abegondo, Lalín, Trazo, Tordoia and Vilalba exhibited vector activity restricted to the summer-autumn period. The remaining analysed locations – Sarria and Viana do Bolo – showed a broader vector activity period, although gaps were still observed during winter (Fig. 2).

**Fig. 3:**
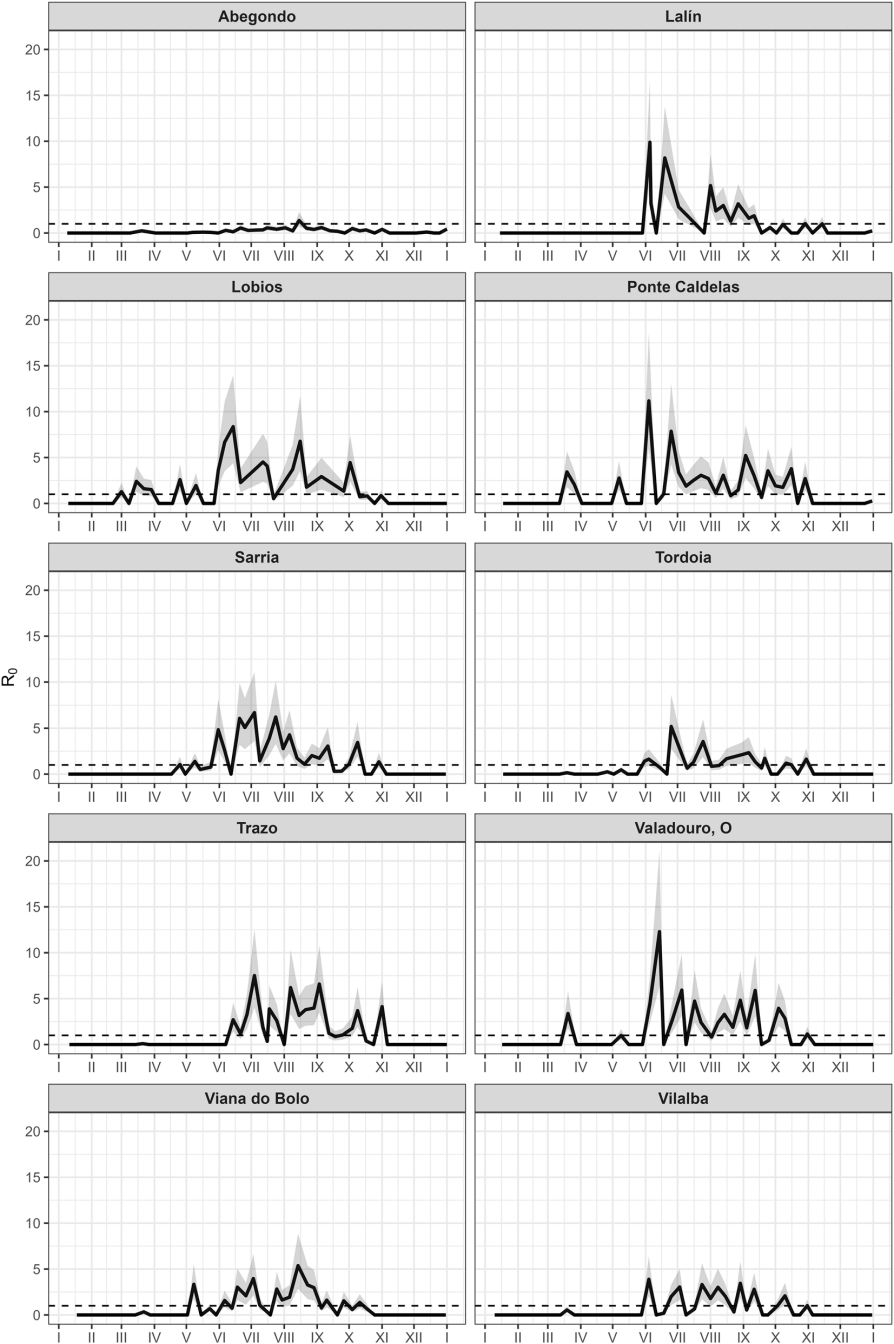
Temporal dynamics of the mean basic reproduction number (*R*_0_) obtained using a Monte Carlo simulation approach, with the 95% uncertainty interval, across the 10 sampling sites during 2009. The dashed line indicates the epidemic threshold (*R*_0_ > 1)

In addition to the population dynamics, the potential BTV transmission cycle was also assessed. Deterministic estimates indicated that the basic reproduction number exceeded the epidemic threshold (*R*_0_ > 1) at least once in every site-by-year combination (Fig. 3). The mean *R*_0_ values obtained from Monte Carlo simulations closely reproduced this pattern, matching the deterministic estimates in 96% of cases. Out of the 500 sampling events, 148 (29.6%) yielded mean *R*_0_ values above the epidemic threshold, most of which were observed during summer. Accordingly, the period of epidemiological risk extended from May to October in most locations, with the exception of Lobios and Ponte Caldelas, where the risk period began as early as April. The highest reproduction numbers were observed in O Valadouro, Ponte Caldelas and Lalín, with maximum values of 12, 11 and 10, respectively (Fig. 3). These values imply 12, 11 and 10 secondary cases, respectively, in a fully susceptible host population generated by a single primary case at the onset of an epidemic. Considering all locations together, *R*_0_ > 1 was estimated for an average of 16.5 ± 7.0 weeks per year (approximately 116 days per year) on the basis of deterministic calculations. This interval was slightly shorter when accounting for parameter uncertainty through Monte Carlo simulations, which yielded an average transmission risk window of 14.8 ± 5.6 weeks per year (approximately 104 days per year).

## Discussion

### Species composition

A comprehensive understanding of the species composition of the *Culicoides* communities across a region constitutes a fundamental element of disease surveillance frameworks and assessments of ecosystem health. *Culicoides obsoletus* s.l. emerged as the predominant species in the study area, consistent with previous findings from research conducted on livestock farms in northern regions of the country [43, 44]. Although morphological discrimination among members of the Obsoletus complex is feasible, it was not undertaken owing to the large number of specimens collected. Nevertheless, Polina et al. demonstrated the predominance of *C. obsoletus* s.s. over *C. scoticus* in a 70:30 ratio in Galicia, based on the examination of male genitalia [20].

Among the potential BTV vectors, *C. punctatus* and *C. pulicaris* were the second and third most abundant species in the study area, respectively. Comparable patterns have been reported in livestock farms in the Basque Country, where the Pulicaris complex constituted the second most abundant taxon and *C. punctatus* the third [44]. *Culicoides newsteadi* was recorded in very low numbers in Galicia, as it is considered a species associated with warmer temperatures, particularly during the autumn season [45, 46].

Only two *C. imicola* specimens were collected in the present study. Their presence may be attributable to passive windborne dispersal from areas where the species is native, as adult individuals are known to be transported over long distance by the wind, or due to the accidental introduction of specimens through inadequately disinfected livestock transports originating from those regions [47, 48].

### Performance of models

Understanding the interplay between the community composition of biting midges and both climatic and non-climatic factors is crucial for determining the risk of vector-borne disease transmission. Accordingly, this study aimed to explain and predict abundance patterns of the most dominant BTV vectors in Galicia in relation to a range of landscape, climate, and host-related variables. The NBGLMM results revealed that these factors play an important role in shaping spatial patterns of local abundance, although the strength of these relationships may vary among species [18, 49].

Considering that the data were obtained in the field, the model showed a good fit for the Obsoletus complex abundance. As previously reported by other authors, temperature plays an important role in the occurrence of this species complex [19, 50, 51]. Specifically, average maximum and minimum temperatures prior to the collection date were identified as key predictors. The average maximum temperature calculated 180 days before sampling had a negative effect, consistent with previous findings showing that extreme temperature values compromise the survival of the taxon [18, 52]. Conversely, the average minimum temperature 28 days prior to collection had a positive influence. After the cold period, during which fourth instar larvae undergo diapause, temperature acts as a signalling mechanism that triggers the end of this phase [51]. Considering that *C. obsoletus* s.l. can complete its life cycle within approximately 1 month under optimal conditions [52], the observed increase in minimum temperature appears to be one of the most influential predictors of abundance.

Another factor that positively influenced the abundance of the complex was the seasonal NDVI, a variable that is itself correlated with soil moisture and vegetation biomass [53]. These findings are consistent with previous studies [18, 53, 54] and align with the known habitat requirements of the Obsoletus complex, which depends on high moisture levels for optimal development and survival [55, 56].

Finally, the last variable influencing *C. obsoletus* s.l. abundance was the season, a categorical factor that has been extensively studied in Spain [57, 58]. Compared with autumn (reference category in the model), the combination of the aforementioned key predictors significantly favoured the proliferation of the species during spring and winter. These results further reinforce the notion that *C. obsoletus* s.l. dynamics respond to a complex interplay among temperature, moisture and vegetation conditions, which jointly define the temporal period of vector activity.

Regarding *C. punctatus*, several studies have identified temperature as a key predictor of its abundance [49, 50, 59]. In the present study, the average maximum temperature 180 days prior to sampling had a negative effect on the species occurrence, supporting the hypothesis that this species is more prevalent in moderate climates [60, 61]. In contrast, average minimum temperature calculated for the 60 days preceding collection showed a positive association with abundance. Although research on the life cycle of *C. punctatus* remains scarce, the increase in minimum temperatures is likely to act in a similar manner to that observed for the Obsoletus complex, both belonging to the subgenus *Avaritia*. In this sense, warmer conditions could serve as a signal for fourth instar larvae to end diapause and resume development.

Although other studies that included altitude have considered it a non-influential factor [62], the models fitted in this study revealed a positive effect of this variable on the abundance of *C. punctatus*. The altitude values recorded ranged from 16 to 934 m.a.s.l., suggesting that this species exhibits a moderately orophilic behaviour within the study area.

The last variable affecting *C. punctatus* abundance was the Köppen Climate Classification, which indicated a significant preference of the species for the Csb-type climate over the Cfb-type. Although both climate types are characterized by mild temperatures in the summer, precipitation is more irregular in Csb regions. The immature stages of *C. punctatus* are frequently found inhabiting organic-rich habitats with a moderate amount of water [63], suggesting that continuous rainfall in Csb climates may limit the availability of suitable breeding sites.

The abundance patterns of *C. pulicaris* were poorly explained by the final model. This may be owing to the limited spatial distribution of available data across the study area, with the exception of some sporadic peaks recorded at specific sites, supporting that this species is more common in southern than in northern Spain [64]. Nevertheless, some preliminary insights can be inferred, as the maximum temperature reached 14 days prior to collection strongly influenced model performance, followed to a lesser extent by altitude. Both variables showed a positive relationship with species abundance, consistent with previous studies identifying these factors as key predictors [45, 50, 65]. In contrast, the model for *Culicoides newsteadi* did not yield conclusive results for any of the variables under study.

Host factors did not show statistical significance for any of the species analysed. No seasonal variation or additional host-related variables were included in the model beyond the officially reported densities of cows, horses and small ruminants, as well as a dichotomous variable indicating the presence or absence of these hosts. *Culicoides obsoletus* is also known to feed on other hosts, such as pigs or even deer [66, 67], but, unfortunately, spatially explicit abundance data for these additional hosts are not available.

Although many of the variables in this study were found to be explanatory of BTV vector abundance, other parameters not considered here could have improved model performance. The most obvious example is the use of insecticides within the study area. Additionally, the availability of suitable biting midge habitats has been shown to be a valuable predictor of their abundance [68]. Therefore, these factors should be taken into account in future research to enhance the predictive power of similar models.

### Phenology and epidemiological risk assessments

The abundance of *Culicoides* spp. vectors in the study area was largely determined by the activity of the Obsoletus complex. Although *C. pulicaris* and especially *C. punctatus* were found in moderate numbers, their presence was characterized by several peaks restricted to specific locations. Specimens belonging to the Obsoletus complex emerged earlier in the year and lasted longer than those of any other taxa, as has been reported in other northern regions of Spain and Europe [57, 69]. The Obsoletus complex typically exhibits bimodal patterns in temperate areas, whereas unimodal patterns are more common in colder regions [70]. Among the 10 sites-by-year combinations analysed, Lobios, Ponte Caldelas and O Valadouro locations displayed two clear abundance peaks. In the remaining cases, either a single peak or a low, constant presence of the species throughout the year was observed. Considering that Galicia is one of the coldest regions of the Iberian Peninsula, the occurrence of two abundance peaks appears to be restricted to situations in which the necessary temperature conditions are met [70]. Although *C. punctatus* is also considered a bivoltine vector [71], its abundance peaks, when present, were recorded during the same periods as those of the Obsoletus complex. This suggests that a constraining factor regarding environmental drivers is taking place, avoiding further proliferation of this species.

The epidemiological assessment of BTV risk across the 10 representative enclaves revealed marked differences between the traditional regulatory metrics and the adapted alternative approach applied in this study. In this way, the mean vector activity period was 23.9 ± 6.0 weeks for the region (around 5–6 months per year). These patterns align with broader observations made in other northern regions of Spain, where vector populations persist throughout spring and autumn and diminish only when temperature compromises their survival [46].

By contrast, the mean period during which the basic reproduction number exceeded the epidemic threshold was estimated at 16.5 ± 7.0 weeks per year based on deterministic calculations, and at 14.8 ± 5.6 weeks per year according to the Monte Carlo adjustment. Despite incorporating parameter uncertainty, Monte Carlo simulations largely reproduced the deterministic estimates, confirming the robustness of the predicted seasonal transmission dynamics. Although simulations slightly reduced the duration of the period during which *R*_0_ values exceeded the epidemic threshold, the overall timing of the transmission season remained largely unchanged. These results suggest that the main conclusions of the model are relatively insensitive to plausible variability in vector-related parameters. Therefore, both deterministic and Monte Carlo approaches indicated a shorter transmission risk window than the VAP, suggesting that periods of vector presence do not uniformly translate into sustained disease transmission potential. The observed discrepancy, with this metric lasting approximately 31.0% (deterministic) to 38.1% (Monte Carlo) shorter than the VAP, highlights the need to incorporate other factors such as the temperature-dependent extrinsic incubation periods, or vector competence dynamics [72]. A clear example is Abegondo, where the VAP lasted 22 weeks, whereas *R*_0_ indicated only one week of epidemiological risk. These results reinforce the utility of *R*_0_ as a complementary tool in a BTV risk assessment. While the VAP/VFP are operationally essential for regulatory compliance, the basic reproduction number enables a more accurate evaluation of the conditions under which BTV could sustain transmission in a susceptible ruminant population [22]. Previous studies have demonstrated that *R*_0_ mapping for BTV can effectively capture spatial and temporal heterogeneity in transmission risk related to both climatic and vector ecology gradients, providing a more reliable epidemiological perspective than data from trap counts alone [22, 36, 73]. These findings also have implications for the optimization of surveillance strategies. Relying solely on trap collections could lead to the overextension of costly control measures outside periods of meaningful *R*_0_ > 1 risk. Conversely, *R*_0_ modelling grounded in local climatic and ecological data identified narrower windows where surveillance and control efforts could be more effectively applied. As a consequence, it appears logical to establish transhumance policies and preventive measures that account for the climatic particularities of the different areas. Nevertheless, for the application of both VAP and *R*_0_ at a local scale, a risk map covering the entire region, rather than only the sampled locations, should be developed [25]. Risk maps based on predictive models, such as those developed in the present study, would be helpful for public administrations. Therefore, this constitutes a valuable next step towards improving entomological and epidemiological surveillance.

## Conclusions

This study contributes significantly to the understanding of the ecological and climatic factors shaping the presence and activity of *Culicoides* species with the potential to transmit BTV in Spain, particularly in its northwestern region. It also represents a pioneering effort in applying both the Vector Activity Period and the basic reproduction number (*R*_0_) index within an Atlantic–Mediterranean context, offering valuable insights into vector dynamics under temperate oceanic conditions.

The approach adopted integrates environmental modelling with vector ecology, enabling a refined framework for assessing potential risk periods. By characterising the interplay between abiotic variables and vector activity, this study provides a solid foundation for improving surveillance strategies and risk-based decision-making. Furthermore, the methodological framework developed herein offers a replicable model for other regions with similar environmental conditions, and serves as a reference for integrating entomological data into vector-borne disease prevention and control efforts.

Although further research is needed to refine predictions and incorporate additional variables, the information provided is of practical relevance for both public and animal health authorities, supporting more effective and targeted intervention policies.

## Data Availability

The data that support the findings of this study are openly available in Zenodo at 10.5281/zenodo.20042883.

## Abbreviations

AIC: Akaike information criterion
AHS: African Horse Sickness
BTV: Bluetongue virus
EHDV: Epizootic haemorrhagic disease virus
IGN: Instituto Geográfico Nacional
KCC: Köppen climate classification
MAPA: Ministry of Agriculture, Fisheries and Food
NBGLMM: Negative binomial generalized linear mixed model
NDVI: Normalized difference vegetation index
NetCDF: Network common data form
PNVLA: Programa Nacional de Vigilancia de la Lengua Azul (referred as Bluetongue National Surveillance Program)
SBV: Schmallenberg virus
VAP: Vector activity period
VFP: Vector free period
VIF: Variance inflation factor
